# Emerging Developments in the Use of Electrospun Fibers and Membranes for Protective Clothing Applications

**DOI:** 10.3390/polym12020492

**Published:** 2020-02-24

**Authors:** Avinash Baji, Komal Agarwal, Sruthi Venugopal Oopath

**Affiliations:** 1 Department of Engineering, School of Engineering and Mathematical Sciences (SEMS), La Trobe University, Bundoora 3086, Victoria, Australia; 20183540@students.latrobe.edu.au; 2 Engineering Product Development (EPD) Pillar, Singapore University of Technology and Design (SUTD), 20 Dover Drive, Singapore 138682, Singapore; komal_agarwal@mymail.sutd.edu.sg

**Keywords:** electrospinning, protective fabrics, textiles, protective clothing, fibers

## Abstract

There has been increased interest to develop protective fabrics and clothing for protecting the wearer from hazards such as chemical, biological, heat, UV, pollutants etc. Protective fabrics have been conventionally developed using a wide variety of techniques. However, these conventional protective fabrics lack breathability. For example, conventional protective fabrics offer good protection against water but have limited ability in removing the water vapor and moisture. Fibers and membranes fabricated using electrospinning have demonstrated tremendous potential to develop protective fabrics and clothing. These fabrics based on electrospun fibers and membranes have the potential to provide thermal comfort to the wearer and protect the wearer from wide variety of environmental hazards. This review highlights the emerging applications of electrospinning for developing such breathable and protective fabrics.

## 1. Introduction

Advancement in nanotechnology has inspired material scientists to explore the use of nanoscale materials and fibers for development of functional and technical textiles [1,2,3,4,5]. Developing advanced textiles and fabrics that offer protection against UV, bacteria, microbes, rain and water is of tremendous interest for personnel working in sectors such as healthcare, agriculture, military etc. [3,4,6,7,8,9]. Traditionally, the materials used for protective clothing applications have been based on impermeable films [10]. Although these materials have shown a considerable amount of success in blocking contaminants and absorbing any penetrated toxins, they are bulky and heavy [10]. Additionally, water vapor transmission through traditional materials is poor. Similarly, impermeable textiles that are based on polymeric films offer good protection but are found to offer poor comfort to the wearer in hot and humid environment. On the other hand, microporous nonwoven membranes have attracted huge amount of interest for the development of protective clothing as they are inexpensive, lightweight and offer excellent protection [3,11]. The air permeability or breathability of these porous nonwoven membranes can be improved by tailoring the pore size within the membranes [4,11].

Recently, electrospinning has gained increased attention for development of protective clothing as it offers the flexibility of controlling the porosity and size of the fibers. Thus, the membranes obtained using electrospinning can display high filtration efficiency and good breathability [2,12]. A straightforward and simple approach of incorporating electrospun fibers into textile involves electrospinning the fibers directly on the surface of the fabric to obtain a composite fabric. This approach of depositing fibers on the surface of the fabric reduces the number of manufacturing steps and at the same time eliminates the issues associated with seam-sealing. Typically, most breathable and waterproof fabrics are composite fabrics that are manufactured by laminating various layers of fabrics together. The electrospun membrane layer can be used to replace the functional layer within these composite fabrics. For example, Vitchuli et al. developed clothing material for protection against chemical and biological warfare agents by electrospinning nylon 6 fibers directly on top of nylon/cotton fabric [13]. Their results demonstrated that the filtration efficiency can be improved by over 250% when electrospun nylon 6 fiber layer is deposited on the fabric. In another study, Lee et al. deposited polyurethane fibers filled with zinc oxide nanoparticles on cotton fabric to develop composite fabric that offers protection against bacteria and UV [4]. Other studies have used a two-step approach to incorporate electrospun fibers into textiles. In the first step, an electrospinning setup is modified to produce yarns. In the second step, these yarns are incorporated into traditional textiles using techniques such as braiding, weaving and knitting for imparting some functionality to the fabric. For example, Wu et al. [14] in the first step, used electrospinning to prepare polyacrylonitrile (PAN) yarns. These yarns are then employed to produce fabrics using techniques such as weaving, knitting and braiding. Their results show great potential of using electrospun yarns for producing protective fabrics. Similarly, Valtera et al. [15] produced composite yarns using electrospinning and used these yarns to manufacture protective fabrics. In their study, a needleless spinneret connected to an AC source is used to generate a plume of nanofibers. These nanofibers are wound around a conventional thread to prepare composite yarns.

This review article presents an overview on the use of electrospun fibers, membranes and yarns for the development of protective fabrics and textiles. These fabrics and textiles incorporated with electrospun fibers have the potential to offer thermal comfort to the wearer and can protect the wearer from wide variety of environmental hazards. This article focuses on presenting various techniques that are used to integrate electrospun fibers, membranes and yarns into the fabrics. The application of these electrospinning enabled fabrics for protection against environmental hazards are also presented. Finally, future perspectives on using electrospinning for developing smart fabrics are discussed.

## 2. Integrating Electrospinning with Textiles

Polymer fibers with submillimeter-to-micron length diameters have often been used in traditional textiles [16,17]. Recently, electrospinning has found increased interest in the fabrication of textiles as the incorporation of nanofibers into traditional textiles can impart some unique functional properties to the textile [2,4,18]. For example, embedding nanofibers in traditional textiles can impart some unique functionalities to the textiles such as self-cleaning, water repellency, and flame-retardancy etc. This section discusses some of the approaches used to incorporate electrospun fibers into textiles and clothing.

### 2.1. Yarns

**Figure 1:**
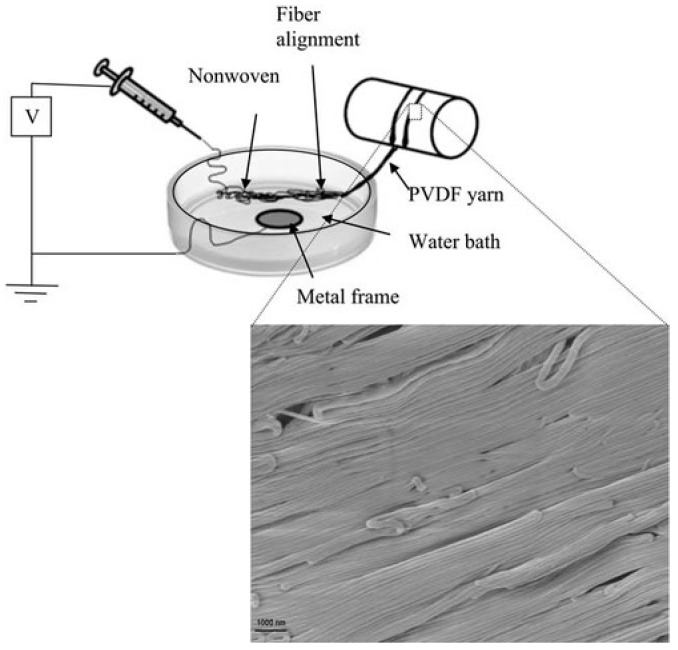
Modified electrospinning setup used to collect linear nanofiber assemblies. Reprinted from *Macromolecular Materials and Engineering*, Vol. 297, Avinash Baji, Yiu-Wing Mai, Xusheng Du, Shing-Chung Wong, Improved tensile strength and ferroelectric phase content of self-assembled polyvinylidene fluoride fiber yarns, 209–213, Copyright (2012), with permission from John Wiley and Sons.

Traditionally, textiles are fabricated using yarns that are drawn from cotton fluffy mass and wound into spools [19]. These yarns consist of micron or macroscale cotton fibers. However, with the advancement in nanoscience, the textile industry is directing its attention to fabricate and incorporate nanofibers within the textiles [20]. It is well understood that single nanofibers are difficult to handle. Hence, they cannot be used directly in weaving or knitting to produce fabrics or textiles. Linear fiber assemblies that are obtained by collecting aligned nanofibers or yarn assemblies that are obtained by twisting the nanofibers ensure that the nanofibers are suitable for weaving or knitting. Over the past decade, researchers have modified the electrospinning setup to produce nanofiber yarns or linear nanofiber assemblies. For example, we have modified the electrospinning setup to collect linear nanofiber assemblies [21,22]. The schematic of the modified electrospinning setup used to collect linear nanofiber assemblies is shown in Figure 1. Here, the fibers are initially deposited on surface of water. They are then drawn out and wound around a mechanical drum. This process led to the alignment of the fibers and enabled us to collect aligned fiber assemblies.

**Figure 2:**
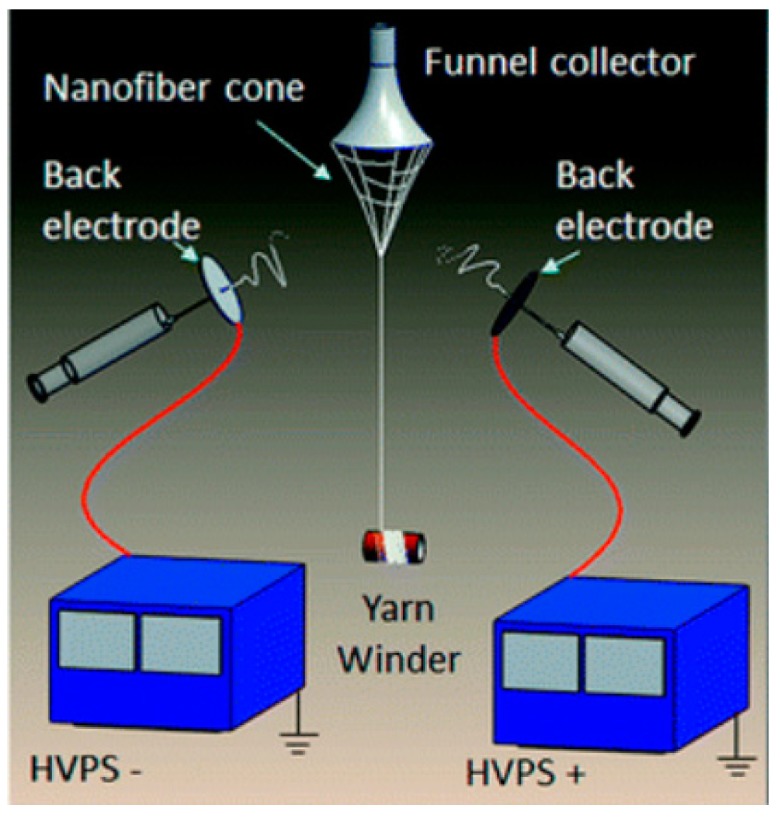
Conjugated electrospinning setup used to produce nanofiber yarns. Reprinted from *RSC Advances*, Vol. 5, Zhigang Xie, Haitao Niu, Tong Lin, Continuous polyacrylonitrile nanofiber yarns: preparation and dry-drawing treatment for carbon nanofiber production, 15147–15153, Copyright (2015), with permission from Royal Society of Chemistry.

Others have obtained nanofiber yarns by using a conjugated electrospinning setup [23,24,25]. Briefly, a polymer solutions are fed to two different spinnerets that are individually connected to positive and negative high voltage power supply. The fibers are spun onto a metal funnel and then drawn out using an insulating rod. The rotation of the metal funnel helps in twisting of the fiber bundles. In the final step, the fiber bundles in the form of yarns are wound around a yarn winder. Figure 2 shows the schematic of the conjugated electrospinning setup used to produce nanofiber yarns. These yarns are then interwoven to form fabrics.

**Figure 3:**
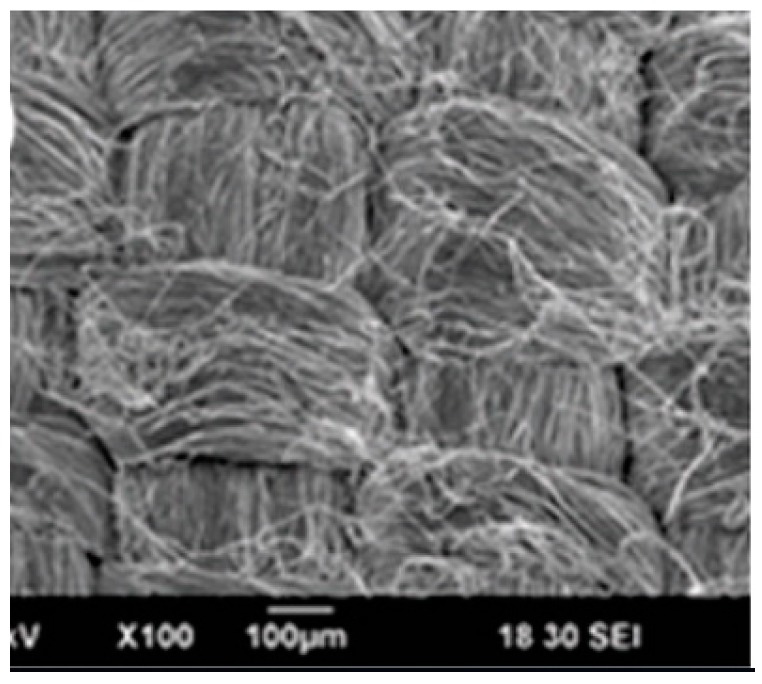
Scanning electron microscope (SEM) image of woven structure obtained using the electrospun yarns. Reprinted with permission from John Joseph, Shantikumar V. Nair, Deepthy Menon, Integrating substrateless electrospinning with textile technology for creating biodegradable three-dimensional structures, *Nano Letters 2015*, Vol 15, 5420–5426. Copyright (2015), American Chemical Society.

A similar setup is used to first produce polyacrylonitrile (PAN) yarns [14]. Following this, a traditional textile machine is used to produce fabrics from the PAN yarns. These PAN yarns are then braided by intertwining three of more yarns to make cylindrical patterns. PAN yarns are also used to produce fabrics with the help of weaving technique. Briefly, warp yarns are pre-stretched across a weaving frame. In the next step, the weft yarns are woven across in the perpendicular direction. Joseph et al. [18] obtained yarns using electrospinning coupled with a substrateless deposition approach. Here, instead of using a funnel, the fibers are electrospun onto a flexible metal electrode placed such that it forms an open-ended frame of a funnel. The fibers are then pulled out as yarns and wound around a yarn winder. Following this, an industrial textile machine is used to ply, braid and weave the yarns to produce various kinds of constructs. They show that open-ended hollow structures can be fabricated by braiding of the yarns. These constructs show tremendous potential for stents and vascular graft applications. Figure 3 shows the scanning electron microscope (SEM) image of the woven construct obtained using these yarns. These woven structures are argued to have tremendous potential for drug-delivering implants.

### 2.2. Nonwovens

Traditionally, nonwoven fabrics are manufactured by expanding the foam until the polymer is converted to fibers [26]. This process is known to produce large quantities of fibers [27]. Although the diameter of the fibers can be around 100 nm, the fibers are mixed with nonfiber phases of polymer. Such membranes have been used to produce functional garments. For example, polyurethane and polytetrafluoroethylene (PTFE) membranes are used to produce wind resistant as well as water resistant garments. An alternative approach based on electrospinning has gained widespread interest to produce nonwoven fabrics as the process is quick, simple and scalable [28,29,30]. The inherent high surface area to volume ratio and small pore size make electrospun nonwoven fabrics suitable for filtration and biomedical applications such as wound dressing and tissue scaffold applications. The porous nature of the nonwoven membranes produced using electrospinning ensure that they provide good resistance against the potentially harmful chemical and biological warfare agents that are in the aerosol form. On the contrary, the membrane is capable of transporting water vapor, which helps in regulating the thermal temperature of the body. Typically, the electrospinning approach is used to deposit nonwoven fabric directly on traditional substrates to develop protective clothing. Using this approach, electrospinning can be used to deposit the fibers in three-dimensional form. Thickness of the nonwoven at various locations on the garment can be varied as desired. This form of producing textiles can eliminate some of the manufacturing steps and could solve some of the issues such as seam-sealing problems that are typically associated with protective garments [31]. Faccini et al. [30] used electrospinning to deposit polyamide 6 (PA6) nonwoven fibrous mat onto a nonwoven viscose substrate to produce protective clothing. A thermoplastic glue followed by hot-press lamination is also used to improve the adhesion between the nanofiber layer and the substrate. Their results show that the developed textiles retained 99% of 20 nm as well as 200 nm size particles. It demonstrates their use for protective clothing applications such as masks and filters. Gibson et al. [32] used electrospinning to produce elastomeric membrane for protective clothing applications. They show that the elastomeric membranes are efficient in trapping air borne particles. They also demonstrated that the gas flow properties of the membrane can be increased by biaxially stretching the membrane. However, the water vapor transport through the membrane remains constant. Their results show that the electrospun membrane can be applied or coated onto the fabrics for protective clothing applications. In a similar study, Lee et al. [3] argue that the surface chemistry of the polymer used for electrospinning should be considered before they are deposited onto the textile material. The surface energy of the material chosen for electrospinning defines the wettability of the electrospun membrane. They used low surface energy material, such as polypropylene, to produce an electrospun membrane. The low surface energy of the polypropylene helped the membrane to repel water and worked as barrier to liquid penetration. In their study, polypropylene fibers are deposited on the textile material using melt electrospinning. The barrier properties and the air/moisture transport properties of the composite material is investigated. They show that although the air permeability and moisture transportation through the laminated protective clothing decreased by ~10% to 20%, they are still found to be higher than most materials used for protective clothing applications. De Falco et al. [5] explored electrofluidodynamic technology (EFD) that is based on electrospinning and electrospraying to coat and modify the surface of textiles. They argued that using this process it is possible to coat large substrates and convert the fabrics into functional fabrics. For example, by depositing conducting graphene or carbon nanotubes on the surface of nonconductive fabrics, the fabric can be made electrically conductive and useful for supercapacitor application. Similarly, by selecting the appropriate coating material, the fabric can be made magnetically or electrically conductive, antibacterial or photo responsive.

**Figure 4:**
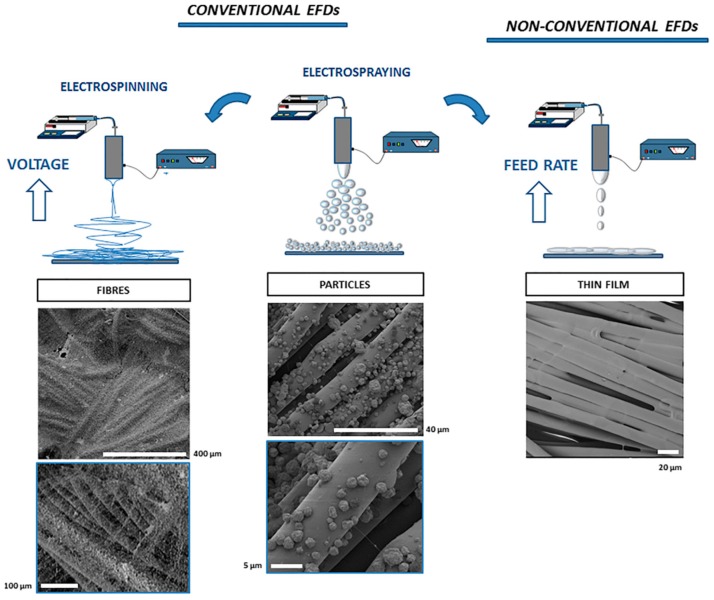
Schematic of the electrofluidodynamic (EFD) process and the corresponding SEM images of the fibers obtained using the EFD process. Reprinted from *Journal of Colloid and Interface Science*, Vol. 541, Francesca De Falco, Vincenzo Guarino, Gennaro Gentile, Mariacristina Cocca, Veronica Ambrogi, Luigi Ambrosio, Maurizio Avella, Design of functional textile coatings via non-conventional electrofluidodynamic processes, 367–375, Copyright (2019), with permission from Elsevier.

Figure 4 shows the schematic of the EFD process and the SEM images of the produced fabrics. The schematic shows that it is possible to deposit either fibers, droplets or thin films on the surface of the fabrics by modifying the EFD processing conditions. They demonstrated that droplets are deposited on the surface of the fabric when the polymer solution has lower content of dissolved polymer. On the other hand, EFD process leads to the formation of fibers when high molecular weight polymer is used to prepare the polymer solution. A thin layer of polymer is deposited on the surface of the fabric by increasing the feed rate during the EFD process.

### 2.3. Composite Fibers and Multilayer Nanofiber Membrane

**Figure 5:**
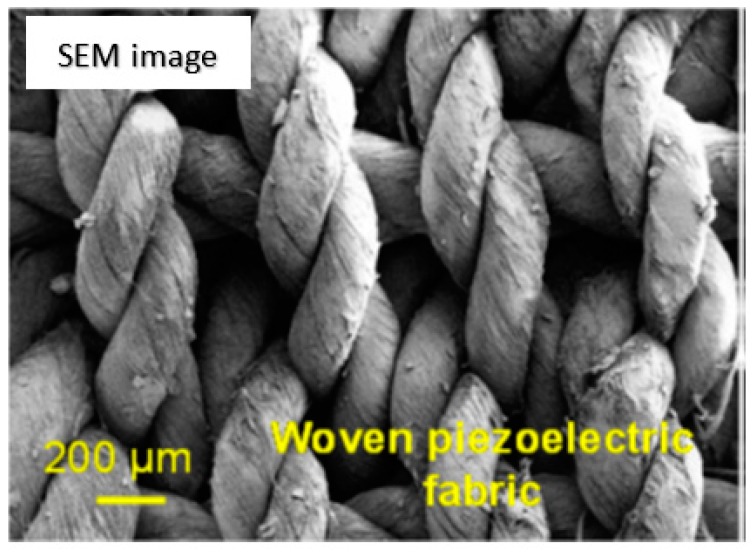
SEM image shows the microstructure of a woven piezoelectric fabric obtained using the plied yarns. Reprinted with permission from Enlong Yang, Zhe Xu, Lucas K. Chur, Ali Behroozfar, Mahmoud Baniasadi, Salvador Moreno, Jiacheng Huang, Jules Gilligan, Majid Minary-Jolandan, Nanofibrous smart fabrics from twisted yarns of electrospun piezopolymer, *ACS Applied Materials and Interfaces 2017*, Vol. 9, 24220–24229. Copyright (2017), American Chemical Society.

In recent years, there has been a considerable amount of interest developed for fabrication of smart and functional textiles. For example, the incorporation of piezoelectric materials within textiles can help the wearer to convert mechanical movements of the body into a form of electric energy. Yang et al. [1] in their study used electrospinning to first fabricate piezoelectric poly(vinylidene fluoride)-*co*-trifluoroethylene (P(VDF-TrFE)) fibers. These fibers are then converted into twisted yarns. Following this, the single yarns are converted to two-ply and three-ply yarns. These yarns are then used as wefts in the woven structure to produce functional fabrics for energy harvesting application. They also fabricated continuous twisted yarns using modified electrospinning setup. Here, the fibers are first collected at the larger end of a funnel. The yarns are then pulled from the thin nanofiber web formed on the funnel and twisted. These twisted yarns are also used to fabricate two-ply and three-ply yarns. Following this, the plied yarns are stitched on a fabric using a sewing machine. The yarns are also used to make woven piezofabric. Figure 5 shows the SEM image of the woven structure produced using the obtained yarns.

**Figure 6:**
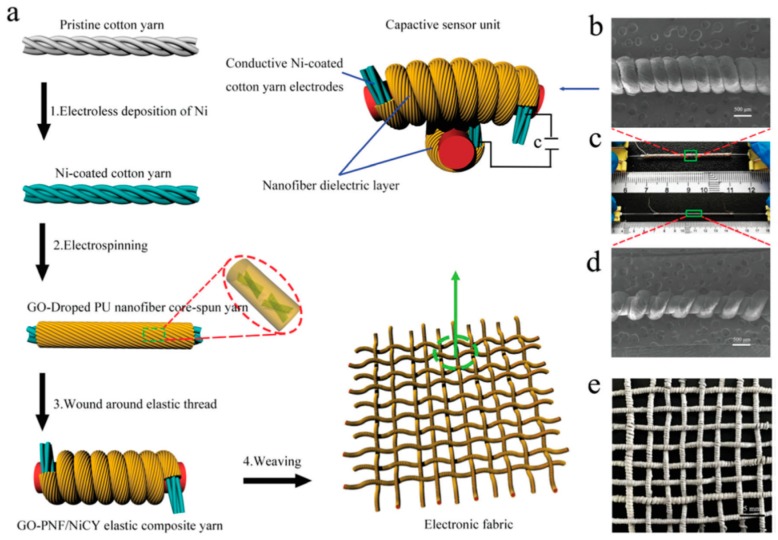
(**a**) Schematic demonstrating the use of yarns for developing electronic fabrics. Briefly, graphene doped polyurethane fibers are deposited on the surface of Ni-coated cotton yarns. These core-shell yarns are then wound around an elastic thread. In the next step, the composite yarns are woven to obtain a fabric. (**b**) SEM image of the elastic composite yarn. (**c**) optical microscopy image of the elastic composite yarn. (**d**) SEM image of the stretched elastic composite yarn. (**e**) optical microscopy image of the woven electronic fabric. Reprinted from *Journal of Materials Chemistry C*, Vol. 6, Xiaolu You, Jianxin He, Nan Nan, Xianqiang Sun, Kun Qi, Yuman Zhou, Weili Shao, Fan Liu, Shozhong Cui, Stretchable capacitive fabric electronic skin woven by electrospun nanofiber coated yarns for detecting tactile and multimodal mechanical stimuli, 12981–12991, Copyright (2018), with permission from The Royal Society of Chemistry.

Using similar approach, textiles for wearable electronic skin applications can be developed by incorporating pressure-sensitive materials into the fabric [33,34,35]. For such applications, functional polymers such as piezoelectric or conductive polymers are typically used and integrated into the fabrics [36,37,38]. Alternative approach is to embed metal nanowires, functional ceramic nanoparticles or carbon nanostructures into the polymer before they are integrated into the fabric. The most common approach involves dispersion of carbon nanotubes (CNTs) or graphene directly into the polymer solution followed by electrospinning to obtain CNT/graphene incorporated polymer fibers [39,40]. The presence of CNT or graphene within the polymer fiber also serves to improve the mechanical strength of the fibers. In the study by You et al. [41], graphene oxide doped polyurethane fibers obtained using electrospinning are wound around nickel coated cotton yarns to form core-spun yarns. The obtained core-spun yarns are then wound around an elastic thread, which is then woven to obtain a fabric. The schematic shown in Figure 6 illustrates the use of yarns for developing electronic fabrics. The cross-over points between the elastic composite yarns serve as capacitive mechanical sensor unit. Thus, when a pressure is applied to the fabric a change in the capacitance is detected. They demonstrated that the sensor unit has excellent pressure sensitivity of 1.59 N^−1^. This electronic fabric shows excellent performance in recording the body posture and movement. Thus, their results demonstrate that these fabrics can potentially be used as artificial skin and wearable health monitoring device.

In another study [42], wearable electronic skin is fabricated by weaving pressure sensitive yarns into a fabric. For this purpose, PVDF yarns are first produced using electrospinning. The obtained PVDF yarns are coated with poly(3,4-ethylenedioxythiophene) (PEDOT) by in situ polymerization of EDOT on the surface of the fiber yarns. The obtained core-shell PVDF-PEDOT yarns are woven into a double-layer wearable fabric. They show that an open-circuit voltage signal is obtained when a pressure is applied. Additionally, the output open-circuit voltage is shown to switch when a pressure is applied. Thus, the fabric is shown to be self-powered under an applied pressure.

## 3. Protective Clothing

### 3.1. Protective Clothing against Micro and Nanoparticles

Recently, there has been an increased concern over exposure to the health and safety risks posed by micro and nanoparticles [13,30]. The presence of micro and nanoparticles in air due to air contamination and air pollution is one of the biggest environmental risks to human health [43]. The particulate matters (PMs) present in air are mixture of organic and inorganic solid and liquid particles. These particles are classified as PM10 and PM2.5, which describes the presence of particles in air with size less than 10 microns and 2.5 microns, respectively [28,44]. Both short-term exposure and long-term exposure to polluted air with high PM10 and PM2.5 can have serious effect on health. It is necessary and important to develop new barrier materials and protective clothing/material against micro/nanoparticles and ensure that the exposure is as low as possible. The conventional air filtration technique relies on using high efficiency particulate air (HEPA) filters that are based on micron-sized fiberglass [45]. Although, these nonwoven filters based on micron-sized fiberglass remove micron-sized particles from the air, they are incapable of removing submicron-sized particles. This limitation is attributed to the presence of a large size of pores present within the nonwoven mesh.

In comparison, filter media produced using electrospun membranes can demonstrate superior performance due to ultrafine size of fibers, huge surface area to volume ratio, large surface energy and presence of fine porosity within the membranes. These attributes of the electrospun membranes ensure that the filter media displays higher filtration efficiency and permeability during the filtration process compared to conventional HEPA filters [46,47,48]. The interplay of various filtering mechanisms is responsible for the separation and filtration of particles from air when electrospun membranes are used as the filter media [28]. For example, particles that are typically in the range of 0.3 to 1 micron in size are separated from the airflow and captured by the fibers due to van der Waals interactions. This occurs when the particles in the airflow come in close contact with the surface of the fibers [49]. Particles that are larger than 1 micron in size get separated from the air flow due to inertial impaction [50]. The airflow through the membrane undergoes a tortuous path. Due to the inertia, the particles collide with the fibers and get deposited on the surface of the fibers. Lastly, particles that are below 0.3 micron in size get separated from the air flow due to electrostatic effect. This occurs when either the particles or the fibers are charged. The electrostatic interaction attracts the particles on to the surface of the fibers [45]. Thus, these separation and filtration mechanisms indicate that the filtration efficiency of the electrospun filter media can be improved by tuning the size of the fibers and their surface chemistry. For example, fibers fabricated using polymers with high dipole moment are shown to capture PM particles with polar functional groups such as C–O, C=O and C–N [51]. This is attributed to the dipole–dipole interaction with the particles and due to induced dipole intermolecular forces that attract the particles to the fibers. It is also reported that the filter’s particle capturing capability is increased when the size of the fibers is reduced to nanometer length scale. Filters with nanometer-sized fibers are found to be transparent to sunlight and they allow sufficient air flow. It has been reported that an electrospun polyacrylonitrile (PAN) membrane with 200-nm diameter fibers can be used as efficient and transparent air filters [51]. These PAN membranes displayed in a field test that they can be used under a hazardous PM2.5 level for ~100 h with efficiency as high as 95%. Strain et al. [52], in their study, developed electrospun membranes based on polyethylene terephthalate (PET) fibers for smoke filtration. They demonstrated that membranes with finer diameter fibers are more effective in smoke filtration compared to membranes with larger diameter fibers. Membranes with 0.4 µm diameter fibers are shown to capture smoke residuals 43 times its own weight. In comparison, a typical cigarette filter tip that is based on cellulose acetate only absorbed smoke residuals 2.7 times in own weight. In an another study [53], Wang et al. developed air filters by depositing electrospun polyvinyl chloride (PVC)/polyurethane (PU) blend fibers on conventional filter paper. They demonstrated that the filter paper with PVC/PU blend fibers possess high filtration efficiency for particulate filtration compared to neat filter paper. Few studies have also fabricated electret fibers to attract and capture electrostatically active nanoparticles in the air [54,55,56,57,58]. Some polymers store electric charges during electrospinning, such as PAN, PVDF, polyamide, and polyetherimide. These electret membranes are shown to be useful as air filters as they attract and adsorb the particles present in the air. It is also demonstrated that the electrostatic property of an electric membrane can be improved by dispersing some charge storage enhancers such as SiO_2_ nanoparticles or nanoscale graphite platelets within the fiber matrix [57,58].

### 3.2. Protective Clothing against Liquid Penetration

One of the important requirements for clothing apart from aesthetics is the function and comfort offered by the clothing to the wearer [12,59,60,61,62]. Protective clothing that is waterproof and breathable can provide thermal comfort to the wearer. It provides the wearer with comfort under extreme climatic conditions and protects them from environmental factors such as rain, snow and wind. This type of clothing restricts the penetration of liquid water while allowing water vapor to transmit through them [11,12,60,63]. Such materials used for clothing can also find applications in wide variety of fields including filter and separation media, medical supplies etc. [62,64].

Typically, materials that offer protection against water display contrasting behavior with water vapor. In other words, water-proof materials are seldom found to be breathable. Recently, it has been demonstrated that waterproof and breathable materials can be fabricated by using porous hydrophobic materials with interconnected passageway of pores [61]. Traditionally, such materials have been fabricated using techniques such as biaxial stretching, phase separation and template-based strategy [65,66,67,68]. However, conventional techniques for fabricating protective materials have obtained limited success in terms of water proof nature of the material and/or its breathable performance. This is attributed to difficulty in regulating and controlling its porous structures.

Recently, researchers have focused their efforts on using electrospinning to make waterproof and breathable materials [63,64]. Electrospinning is capable of fabricating membranes that are not only densely packed but also have interconnected pores. The presence of micron and nano sized pores in the electrospun membrane allows the transport of moisture and restricts the entry of water and wind. However, it is reported that the electrospun materials demonstrate good waterproof and breathability only when they are fabricated using an inherently hydrophobic polymer [61]. Electrospun membranes fabricated using hydrophobic polymers resist the entry of water droplets and transmit the water vapor molecules.

**Figure 7:**
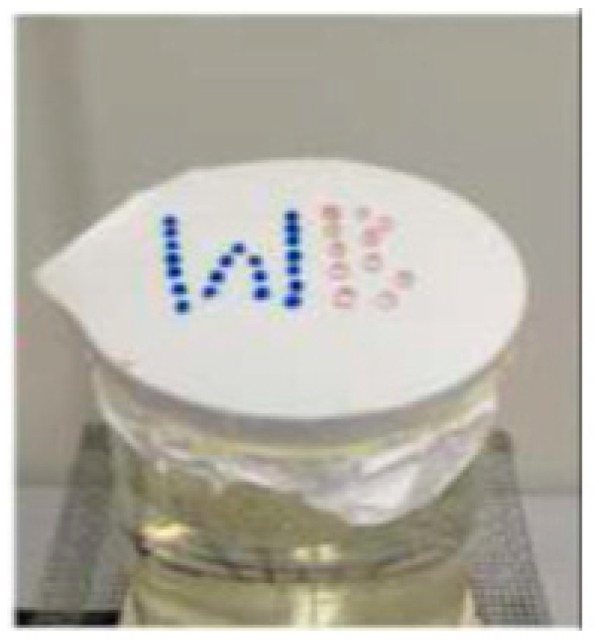
Waterproof and breathability investigation of an electrospun PAN-modified PDMS membrane. The electrospun PAN modified PDMS membrane is made to cover the beaker with water. Due to the hydrophobicity of the membrane, the water droplets placed on the membrane are restricted from entering the membrane. On the other hand, when the setup is heated, the membrane is permeable to water vapor. Thus, when silica gel is placed on the membrane, it changes its color from blue to pink. Reprinted with permission from Junlu Sheng, Min Zhang, Yue Xu, Jianyong Yu, Bin Ding, Tailoring water-resistant and breathable performance of polyacrylonitrile nanofibrous membranes modified by polydimethylsiloxane, *ACS Applied Materials and Interfaces 2016*, Vol. 8, 27218–27226. Copyright (2016), American Chemical Society.

Kang et al. [11] used electrospinning to produce waterproof and breathable fabrics based on polyurethane fibers. In their study, they used electrospinning to deposit polyurethane fibers onto a substrate fabric to produce breathable fabrics. They also produced control samples by coating polyurethane resin membranes on the fabrics. They demonstrated that the waterproof and breathability performance of fabrics based on electrospun polyurethane fibers is much higher compared to the control samples. Sheng et al. [60] used electrospinning to first produce a polyacrylonitrile (PAN) nanofibrous membrane and then modified the membrane by adding polydimethylsiloxane (PDMS) to the membrane. They demonstrate that neat PAN nanofibers are hydrophilic. However, PAN modified PDMS membranes are hydrophobic and demonstrate low contact angle hysteresis. Water droplet is shown to roll off from the surface of the PDMS modified PAN membrane when it is tilted by 7°. They also demonstrated that due to fine interconnected pores these membranes are permeable to water vapor. Figure 7 illustrates the breathability mechanism of their membrane. The beaker filled with water is covered with the membrane and the setup is heated to 100 °C for 30 min. Water stained with a blue dye is shown to rest on the surface of the membrane. However, the silica gel placed on the surface of the membrane is shown to change color from blue to pink. This shows that the membrane is permeable to water vapor.

The performance of the electrospun membrane in terms of its breathability can be enhanced by controlling its pore size and overall porosity. A reduction in pore size will play an enhanced role in resisting the entry of the water droplets. On the other hand, improving the overall porosity in the material will increase the transmission of water vapor out of the material. Li et al. [69], in their study, investigated the effect of the pore size, porosity and pore length on the electrospun membrane’s breathability and waterproof performance. They used electrospinning to prepare membranes based on polyurethane and fluorinated polyurethane. The porosity and pore size of the membranes is tailored by controlling the relative humidity during the electrospinning process and the duration of the electrospinning. The breathability of the membranes is investigated covering a beaker of water with the fabricated electrospun membrane. They demonstrated that when the beaker is heated, an abundant amount of water vapor is generated and transmitted through the membrane. The breathability is attributed to the Fickian diffusion of water vapor through the porous membrane. This is driven by the difference of relative humidity between the two sides of the membrane. The breathability of the membranes is shown to depend on the porosity and pore length. More recently, Shi et al. [70] fabricated a Janus membrane using electrospinning and demonstrated that water penetrated the membrane from only one surface of the membrane. The Janus membranes are produced by the fabrication of a bilayer structure with one hydrophilic layer and the other being hydrophobic. Water is shown to penetrate when it is placed on the hydrophobic side of the bilayer structure. In our recent study, we fabricated Janus membrane using electrospinning [71]. We demonstrated that water penetrates when placed on the hydrophobic side of the Janus membrane but does not penetrate when placed on the hydrophilic side of the Janus membrane. These Janus membranes show tremendous potential for developing breathable and waterproof fabrics.

### 3.3. Protective Clothing against Chemicals 

The development of clothing for protection against chemical warfare agents utilizing innovative protective systems is of utmost importance to the military and security personnel. Chemical warfare agents such as chlorine, arsine, phosgene etc., are considered the most nefarious chemicals as they damage the skin and nerves [72,73]. Conventionally, protective textile systems are based on the usage of multilayered fabric with a protective layer [2,13,74,75]. The protective layer in these fabrics consist of inorganic material such as activated carbon or activated charcoal, which helps in capturing the chemicals and preventing them from penetrating the fabric. Although, these conventional protective fabrics have shown tremendous success in capturing the chemicals, they have a high thermal burden and are not permeable to air and water vapor. The lack of breathability of the conventional chemical protective textile causes discomfort to the wearer and reduces their efficiency in performing active tasks. The other disadvantage associated with convention protective clothing is the disposal of the used or contaminated clothing. The conventional protective clothing is only capable of physically adsorbing the chemical agents. They do not help in detoxifying the chemical warfare agents. Some metal oxides have been identified as an alternative to activated carbon or charcoal in protective clothing. The metal oxides are shown to have superior reactivity to chemical warfare agents and are also capable of detoxifying the chemical agents [76]. Recently, electrospinning is used to fabricate these inorganic metal oxide fibers that can be incorporated into chemical protective clothing. The electrospun fibrous membranes resist the permeation of organic molecules but are permeable to air and water vapor.

Vitchuli et al. [74] used electrospraying along with electrospinning to prepare nylon 6 fibers coated with zinc oxide (ZnO) nanoparticles. They demonstrated that these fibers have good detoxifying efficiency against paraoxon, which is a simulant of highly toxic organophosphorus compound. To demonstrate the detoxification efficiency of the ZnO coated nylon 6 fibers, the nanofiber membrane is placed in a glass vial filled with a solution of paraoxon. After specific time duration, the solutions are tested using gas chromatography-mass spectrometry and the residual concentration of paraoxon in the solution is determined. Their results show that the paraoxon concentration decreased significantly in solution that was exposed to the ZnO/nylon 6 nanofibers compared to the virgin untreated solution and to the solution that was exposed to neat nylon 6 nanofibers. The nanofibers presented a large surface area for the absorption of paraoxon. Additionally, the presence of the ZnO nanoparticles on the surface of the fibers helped in the detoxifying the paraoxon molecules by breaking the P–O bonds. The detoxification resulted in the formation of less harmful nitrophenol. Similar study by Ramaseshan et al. [72] showed that electrospun zinc titanate nanofibers can be used as reactive sorbents to detoxify harmful simulants of nerve and mustard agents. The zinc titanate nanofibers are produced using the sol-gel combined with electrospinning technique, which is commonly used to produce ceramic fibers [77,78]. Their results show that the fabricated inorganic nanofibers are porous and have a high surface area. These attributes of the nanofiber ensured that the warfare agents are not only adsorbed on the surface of the fibers but are also decomposed into nontoxic products. They claim that such materials can be used in face masks and for other protective clothing applications.

### 3.4. Protective Clothing against Microbes

Fabrication of cost-effective antimicrobial materials are of great interest for the development of antimicrobial protective clothing. Typically, metals such as silver, copper, cadmium etc. are used for antimicrobial applications due to its ability to deactivate the bacteria [79]. Among all these elements, silver is demonstrated to be most toxic to microorganisms and hence it has been widely used in applications such as antimicrobial filters, wound dressing materials, water treatment, air filtration and protective clothing [80]. It is argued that the electrostatic interactions between the bacterial cells and the silver ions is the main mechanism behind the bactericidal property of silver. Silver cation binds to sulphur, oxygen and nitrogen groups as they are the electron donor groups. This interaction brings structural changes in the cell that leads to rupturing of the cell wall and death of the bacterial cell [81]. Silver ions also tend to bind to bacterial DNA and RNA by denaturing. Due to this antimicrobial behavior of silver, researchers have dispersed silver particles in the electrospinning solution to produce antimicrobial electrospun fibers. Silver particles are either embedded into the fiber matrix or coated on the surface of the fibers. The small diameter of the fibers and the nanometer size of the silver particles ensures that the fibers offer enormous surface area per unit volume. This helps to increase the antimicrobial efficiency of silver coated fibers.

Quiros et al. [81] dispersed silver in polyvinylpyrrolidone (PVP) solution and electrospun this solution to obtain antimicrobial fibers. Silver is shown to reduce and form nanoparticles during electrospinning. The efficiency of these fibers against the bacteria *Escherichia coli* and *Staphylococcus aureus* is investigated. These fibers are shown to decrease the metabolic activity and cell viability. Clear inhibition of the microorganisms in contact with the fibers is demonstrated. They also demonstrated antibacterial effect of the silver-loaded PVP fibers. In a similar study [82], the authors dispersed silver ions in a dimethyl formamide (DMF) solvent to reduce the ions to silver nanoparticles. Following this, polyacrylonitrile (PAN) is dissolved in the DMF solution with silver nanoparticles and electrospun. Irradiating the obtained fibers in UV light is shown to expedite the silver nanoparticle formation. The resultant fibers are shown to possess good bactericidal effect. Their results present a case for using these fibers as filters to protect the personnel from bacterial contamination. Similar approach of reducing silver to silver ions has been reported by other studies [80,83]. For example, ascorbyl palmitate, which is a derivative of vitamin C is employed to reduce silver ions to silver nanoparticles. Ascorbyl palmitate is added to the poly(*ε*-caprolactone) (PCL) solution. Silver ions are dispersed in this solution before the solution is electrospun to produce fibers. The presence of ascrobyl palmitate in the fibers is shown to reduce the silver ions to silver nanoparticles. In another study [80], PVP is used as a capping agent in the formation of silver nanoparticles on the surface of the electrospun fibers. PVP is shown to have strong interaction with the surface of the silver nanoparticles as it bonds with the O and N atoms. Thus, adding PVP to the electrospinning solution helped in reducing the silver ions to silver nanoparticles. The presence of PVP also helps to disperse silver as it prevents the aggregation of the silver nanoparticles on the fibers. Uniform dispersion of silver plays a role in improving the antimicrobial behavior of the fibers.

**Figure 8:**
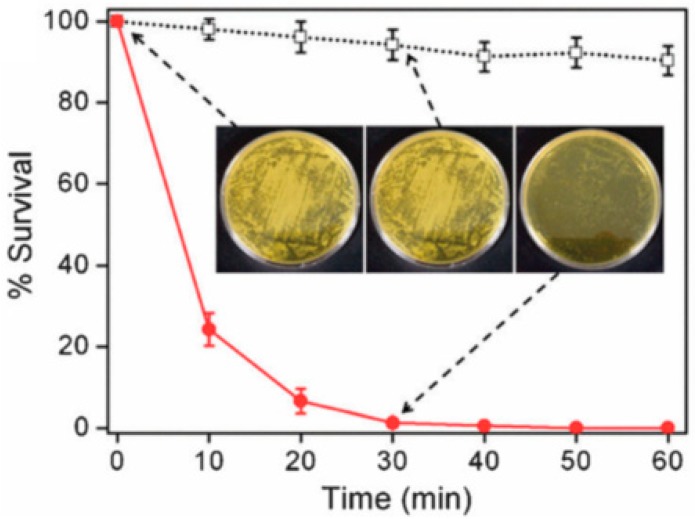
Plot of % survival of bacterial colonies against time for ZnO/TiO_2_ composite (red dots) fibers and TiO_2_ fibers (black hollow squares) against Staphylococcus aureus in the absence of light. The inset demonstrates the bacterial colonies. Reprinted from *Chemical Communications*, Vol. 47, Sun Hye Hwang, Jooyoung Song, Yujung Jung, O. Young Kweon, Hee Song, Jyongsik Jang, Electrospun ZnO/TiO_2_ composite nanofibers as a bactericidal agent, 9164–9166, Copyright (2011), with permission from The Royal Society of Chemistry.

Hwang et al. [84] used an electrospinning technique to fabricate ZnO/TiO_2_ composite fibers and demonstrated their antimicrobial activity against *Esherichia coli* and Gram-positive *Staphylococcus aureus*. The authors also fabricated neat TiO_2_ fibers and compared the antibacterial activity of these fibers with the antibacterial activity of ZnO/TiO_2_ composite fibers. Their results show that when UV is not irradiated, neat TiO_2_ fibers only moderately inhibited bacterial cell growth. On the other hand, ZnO/TiO_2_ composite fibers are shown to effectively kill both *Esherichia coli* and gram-positive *Staphylococcus aureus.* This is attributed to the hydroxyl radicals on the surface of the composite fibers. These reactive hydroxyl radicals are formed due to the presence of ZnO within the composite fibers. The hydroxyl radicals are responsible for killing the cell as they can react with DNA, cell membranes and cellular proteins. Figure 8 shows the antibacterial activities of the composite fiber and TiO_2_ fiber against gram-positive *Staphylococcus aureus*. Figure 8 shows that the % survival of the bacterial colonies are drastically reduced for the composite fibers. These materials show tremendous potential for developing antimicrobial fabrics.

### 3.5. Protective Clothing against Heat and Thermal

In recent years, there has been a growing demand for the development of thermal protective clothing to protect the wearer from different types of thermal exposures such as heat, radiant heat and hot surfaces [85,86]. In conventional thermal protective fabrics, the fabric comprises of two major phases, which influence the thermal characteristics of the protective clothing [87]. The gaseous phase present within the pores of the fabrics consist of water vapor and/or dry air. On the other hand, the solid phase consists of the fiber yarns. These yarns within the nonwoven or woven fabric help to trap the air in the gaseous phase of the fabrics [87]. The gaseous phase within the fabric transfers lesser amount of thermal energy through the fabrics compared to the solid phase. Thus, insulation characteristics of these conventional fabrics is influenced by the yarn properties.

**Figure 9:**
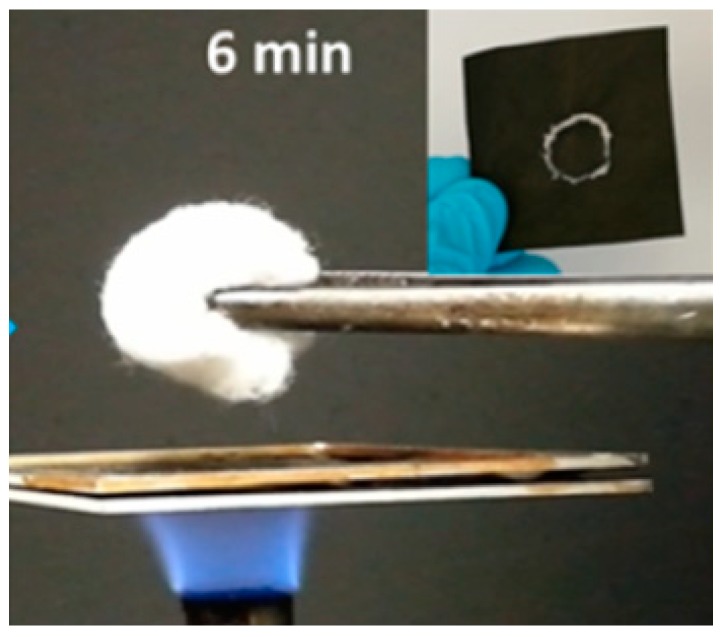
Digital photograph demonstrating the flame-retardant ability of the Hec/BBB membrane. Hec/BBB membrane is brought in contact with a flame and a cotton ball is held on the other side of the membrane. The membrane is demonstrated to block the flame and protect the cotton ball. Reprinted with permission from Jian Zhu, Josef Breu, Haoqing Hou, Andreas Greiner, Seema Agarwal, Gradient-structured nonflammable flexible polymer membranes, *ACS Applied Materials and Interfaces 2019*, Vol. 11, 11876–11883. Copyright (2019), American Chemical Society.

Kevlar fibers and aromatic polyamide, Nomex^®^ fibers due to their flame-retardant characteristics show tremendous potential for developing thermal protective fabrics [88]. Similarly, polymers such as polyimide, polybenzimidazole and polyamide have high degradation temperature and have self-extinguishing ability due to their rigid backbones and chain-to-chain interactions [85]. Membranes fabricated using these high performance polymers offer several advantages such as breathability and heat resistance [86]. Thus, if electrospinning is used to produce nonwoven membranes using these high-performance polymers then they can also be used for thermal protective clothing applications. Zhu et al. [85] used electrospinning to prepare composite materials based on polybisbenzimidazobenzophenanthroline-dione (BBB) and demonstrated that these materials do not sustain a flame or burn. BBB fibers are obtained by first preparing the precursor solution that consists of diaminobenzidine and 1,4,5,8-napththalene tetracarboxylic acid (NTCA). PVP solution is then added to this solution and finally the solution is electrospun to obtain precursor fibers. BBB fibers are obtained by heat treating these precursor fibers. Following this, the obtained BBB fibers are mechanically chopped to produce short BBB fibers. These short BBB fibers are dispersed in sodium hectorite (Hec) solution. Hec and BBB is then deposited on the thermoplastic polyurethane (TPU) membrane. In the following step, the TPU membrane is selectively etched out to obtain Hec/BBB composite fibers that do not ignite when in contact with flame. They argue that the Hec nanosheets in the composite forms a layer and hinders the volatiles from passing through the membrane. The heat transfer across the membrane is retarded. The authors [85] demonstrated that BBB polymer pyrolyzes when neat BBB membrane is brought in contact with flame. On the other hand, Hec/BBB membrane is shown to sustain contact with the flame. Figure 9 shows the setup used to demonstrate the thermal stability and flame-retardancy of the Hec/BBB membrane. The membrane is shown to shield and protect a cotton ball from the flame.

Hang et al. [89] obtained melamine fibers by electrospinning melamine formaldehyde resin and demonstrate its application for developing heat resistant fabrics. They show that the decomposition temperature of the fibers is higher than that of most polymer fibers. Serbezeanu et al. [86] synthesized polyimide (PI) and electrospun the PI fibers onto Kevlar substrates. This material is compared with the conventional thermal protective clothing and demonstrated to have improved protection and water vapor transmission rate. Their material is shown to have good protection against heat and flame.

### 3.6. Protective Clothing against Ultraviolet (UV) Radiation

In recent years, UV-blocking textiles and fabrics have gained significant interest for protection against terrestrial UV radiation [90]. It is well known that the exposure to the sun and the UV radiation can cause significant damage to the skin. For example, exposure to the UV radiation with the wavelengths in the range of 315–400 nm produces the signs of aging [91,92]. On the other hand, exposure to UV radiation with wavelengths in the range of 280–315 nm is responsible for the development of skin cancers [91,92]. Thus, it is crucial for the development of UV blocking textiles particularly for personnel working outdoors for prolonged period. Traditionally, zinc oxide (ZnO) has been used in fabrics and personal care products such as sunscreens due to their ability to block UV radiation [93,94,95]. The effectiveness of these zinc oxide particles to block UV radiation can be enhanced if they are in the nanoparticle form. This is attributed to the increase in the surface area to volume ratio. Lee et al. [4] developed UV-protective textiles by first dispersing ZnO nanoparticles in polyurethane solution before using electrospinning to produce polyurethane filled ZnO fibers. These fibers are deposited onto a polypropylene nonwoven substrate to demonstrate that a very thin layer of polyurethane filled ZnO fibers successfully reduces the UV transmission for both UV-A and UV-B. The presence of ZnO in the fibers helped in UV absorption and ensured that the electrospun fibers are effective in shielding UV radiation. They also show that the UV blocking ability of fibers increased when the concentration of ZnO in the fibers increased and when the density of the polyurethane filled ZnO fibers is increased. The fabricated fibers can reduce the UV radiation by over 97%. In comparison, the untreated substrate offered limited or no protection against UV radiation. Pant et al. [96] prepared UV-protective textile by dispersing titanium dioxide nanoparticles in electrospun fibers. They also reported that the presence of titanium dioxide particles in fibers block UV radiation. This is attributed to the absorbance of UV light by titanium dioxide particles. The absorbance of the UV light is responsible for transferring the electron from the valence band to the conduction band and helps to block UV radiation. In a similar study, Li et al. [92] used electrospinning techniques to produce nanofibers based on natural cotton cellulose. These fibers are then functionalized with cerium dioxide (CeO_2_) using hydrothermal method and their UV blocking capabilities are explored. They demonstrated that the natural cotton cellulose fibers that are modified with cerium dioxide nanoparticles can block UV radiation compared to neat fibers. Cerium dioxide is argued to be sensitive to light absorption and particularly to the absorption bands in the UV domain. Additionally, the large refractive index of cerium dioxide and its uniform distribution on the surface of the fibers played a role in efficient UV scattering. Recently, Lee et al. [97] demonstrated that organic fibers based on lignin and poly(vinyl alcohol) (PVA) are capable of blocking UV radiation. Briefly, lignin and PVA are dissolved in a solvent and electrospun to obtain lignin/PVA fibers. The fibers are cross-linked and their ability to block UV radiation is examined. The transmittance of UV-A and UV-B radiation through the lignin/PVA fibers is shown to be almost zero. Here, the UV-absorbing functional groups such as phenols, ketones and chromophores present in lignin ensure that it can block the UV radiation.

## 4. Future Perspectives

The use of electrospinning to produce fabrics and textiles can bring about a wave of revolution for protective clothing applications. For example, the widespread use of nanoparticles and nanomaterials has raised some serious questions as exposure to these materials can pose a risk to the health and safety. To make matters worse, number of people getting exposed to these nanomaterials are going to increase in future. Thus, there is an urgent need for the development of novel barrier materials against these nanomaterials. The use of electrospun membranes has shown tremendous promise and potential as protective media against the micron-sized particles. The efficiency of the electrospun membrane to capture micron-sized particles can be further improved by controlling the size of the fibers, size of the pores and overall porosity within the membrane. However, their ability to capture particles smaller than 100 nm is seldom discussed. One approach to capture the nanoparticles effectively is via electrostatic interaction. For this purpose, the membrane needs to function as an electret to attract the nanoparticles. Alternatively, the surface of the fibers can be functionalized to promote chemical interaction between the nanoparticles and the fibers. Thus, the membrane will be useful to capture not only the microparticles, but will also be capable of capturing the nanoparticles. In addition to this, the surfaces of the fibers can also be functionalized with additives to capture the pollutants present in the air such as volatile organic compounds, chemical and biological pollutants and convert them into simple compounds. Fabrication of such membranes will have a huge impact on development of next generation of protective air filter membranes. Similarly, membranes that resist the entry of water but transmit water vapor show tremendous potential for developing breathable protective fabrics. Such fabrics can be used for wide variety of applications such as clothing, shoes, separator media, filtration etc.

Similarly, functional and smart fabrics can be produced by combining two different materials and then electrospinning can be used to obtain composite fibers. These fibers can then be incorporated into a clothing membrane to obtain smart and functional fabrics. The fibers can be directly electrospun onto a fabric. Although, this approach eliminates the issues related to seaming the electrospun membrane and fabric together, the adhesion between the membrane and fabric remains a challenge. Plasma treatment and chemical additive methods are being explored by few research groups to improve the adhesion between the membrane and the fabric. Electrospinning can also be used to disperse inorganic antimicrobial particles and agents within and on the surface of polymer fibers. When incorporated into the clothing, these fibers can impart antimicrobial properties to the clothing. Thus, electrospinning offers huge potential for developing membranes and fibers for protective clothing applications.
